# A Case of Tuberous Sclerosis Without Multiorgan Involvement

**DOI:** 10.5539/gjhs.v7n5p124

**Published:** 2015-02-24

**Authors:** Parisa Falsafi, Ali Taghavi-Zenouz, Reza Khorshidi-Khiyavi, Nariman Nezami, Mehrdad Asghari Estiar

**Affiliations:** 1 Department of Oral Medicine, School of Dentistry, Tabriz University of Medical Sciences, Tabriz, Iran; 2 Department of Oral and Maxillofacial Surgery, Faculty of Dentistry, Tabriz University of Medical Sciences, Tabriz, Iran; 3 The Russell H. Morgan Department of Radiology and Radiological Sciences, the Johns Hopkins Hospital, Baltimore, MD, USA; 4 Department of Medical Genetics, School of Medicine, Tehran University of Medical Sciences, Tehran, Iran

**Keywords:** tuberous sclerosis, tubers, nodules

## Abstract

Tuberous sclerosis or Tuberous sclerosis complex (TSC) is a relatively rare autosomal dominant and progressive neurocutaneous disorder involves multiple organs mainly brain, heart, kidney, lung, liver, skin and eye. The diagnosis is typically made clinically. Here, we are reporting a case of TSC presented mainly with dermatologic findings and only neurologic manifestations on MRI. A 15-year-old female with intellectual disability is followed up at neurology clinic for history of seizure. Intelligence evaluation showed that she has intellectual disability. She had wart like lesions distributed in form of butterfly over the face especially involving nose. She did not have any sign and symptom of heart, kidney, lung, bone and eye involvement. Also, her laboratory tests were normal. Despite the physical examination showed absolutely intact neurologic examination, but brain MRI and CT scan revealed several cortical and subcortical tubers, and subependymal glial nodules; no evidence of giant cell astrocytomas and aneurysm. Hypesignal foci are seen at subcortical white matter on long TR images. Fibers are involved. In this case, there is no evidence of giant cell astrocytomas and aneurysm. It seems that TSC could be the prevalent disorder and referring intellectual disability patients in birth with normal organs could be diagnosed as TSC. Therefore, there is necessary need to design genetic natal and post natal tests for diagnosis of TSC cases. Also, there is pivotal that similar cases must be reported; perhaps TSC is more prevalent than to be considered.

## 1. Introduction

Tuberous sclerosis or Tuberous sclerosis complex (TSC) is a progressive neurocutaneous disorder involves multiple organs mainly brain, heart, kidney, lung, liver, skin and eye (Atalay et al., 2010; Leung & Robson, 2007; Curatolo et al., 2008; Franz et al., 2010). In brief, heterozygous mutations in the *TSC1* or *TSC2* genes cause this autosomal dominant genetic disorder (Fombonne, 2003; Smalley, 1998; Smalley et al., 1992). The diagnosis is typically made clinically. The manifestation of the disease is highly variable (Leung & Robson, 2007; Curatolo et al., 2008; Franz et al., 2010) from skin lesions in more than 90%, to 90% of cerebral involvement, 70-90% renal abnormalities, about 50% of retinal hamartomas (Curatolo et al., 2008), and the characteristic rhabdomyoma in 40%-60% of patients (Madueme & Hinton, 2011). Patients could present with oral lesions range from dental enamel pitting (48%-100%), gingival fibromas (50%), fibrous hyperplasia, bifid uvula, haemangioma, high-arched palate, cleft lip and palate, macroglossia, thickening of the alveolar bone, to pseudocystic lesions of the mandible (Leung & Robson, 2007; Schwartz et al., 2007). Commonly, renal complications are the frequent cause of mortality in TSC patients and Lymphangioleiomyomatosis (LAM) is the next common cause of TSC-related mortality (Franz et al., 2010). Here, we are reporting a case of TSC presented mainly with dermatologic findings and only neurologic manifestations on MRI.

## 2. Case Report

**Figure 1:**
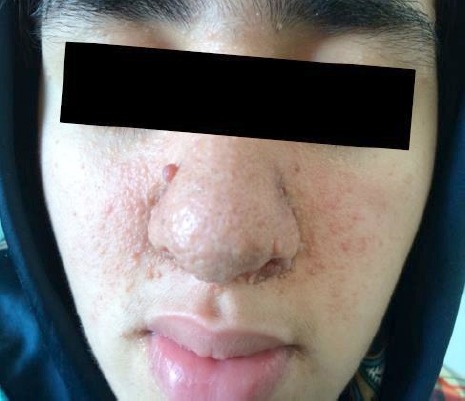
There are extensive wart–like lesions (facial angiofibroma) in a butterfly distribution over the face

A 15-year-old female with intellectual disability, who was referred to the Tabriz Dentistry Hospital with chief complaint of dental caries and tooth pain. She is followed up at neurology clinic for history of seizure and takes Carbamazepine and Phenobarbital. Intelligence evaluation showed that she has intellectual disability. She had wart like lesions distributed in form of butterfly over the face especially involving nose (Figure 1). She started to develop these wart like lesions on her face at the age 7, and the large one measures 0.5 cm in diameter now.

**Figure 2:**
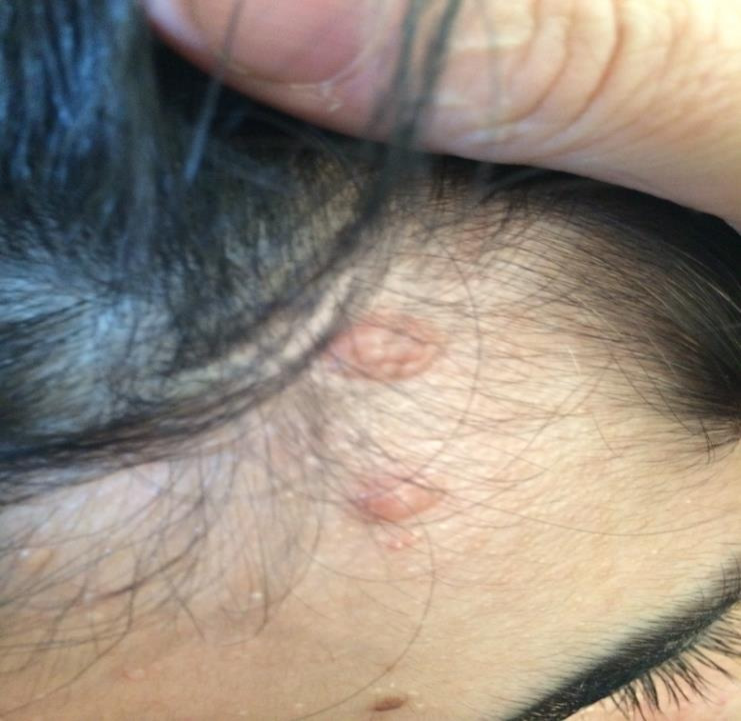
The popular, sessile, flat surface, firm consistency, 1×1×1cm dimension lesions (Fibromas) also located inner folds of hairs

**Figure 3:**
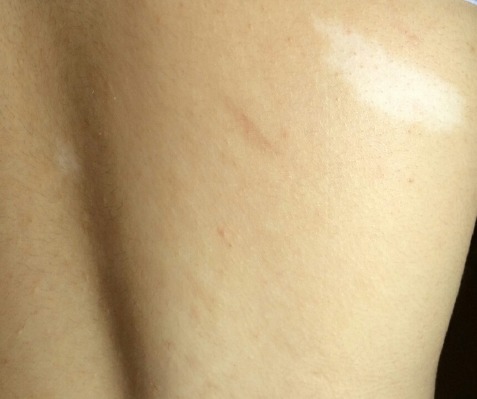
Hypomelanotic lesions (ash leaf spot) on skin

Furthermore, multiple fibromatous lesions characterized with popular, sessile, flat, firm consistency, sized 1×1×1cm dimension are also found in inner folds of hair (Figure 2). She had 2×2 cm hypomelanotic lesions (ash leaf spot) on her back (Figure 3). The lesions were not tender and didn’t tend to bleed.

She did not have any sign and symptom of heart, kidney, lung, bone and eye involvement such as retinal hamartomas, micronodular multifocal pneumocyte hyperplasia, cardiac rhabdomyomas, lymphangioleiomyomatosis, angiomyolipomas, uterine leiomyomas and sclerotic bone lesions. Also, her laboratory tests were normal (Table 1).

**Table 1:** Laboratory tests of TS case

Tests	Results	Units	Normal Range
**Hematology**			
**Hematology tests**			
Bleeding Time-BT	1.10	Min	1-6
PT-Patient-Time	13	Sec	12-13
PT-Ratio	1	%	
INR	1	2-3
PT-Control	13	Sec	13
PTT	32	Sec	28-45
Blood Group (ABO)	A	
Rh Factor	Positive	
**WBC**			
Lymph	54.1	%	25-45
Mixed	6.6	%	4.1-8.1
Neut	39.3	%	45-75
Lymph	3.57	X1000/mm3	1.1-4.8
Mixed	0.44	X1000/mm3	0.2-0.8
Neut	2.59	X1000/mm3	1.8-7.8
**RBC**		
Hgb	12.3	g/dl	12-16
HCT	38.5	%	36-51
MCV	92.5	Fl	77-94
MCH	29.6	Pg	26-33
MCHC	31.9	g/dl	31-37
RDW-CV	13.7	%	11-16
RDW-SD	48.1	Fl	39-46
**PLT**			
PDW	9.1	Fl	7.1-20
MPV	8.2	Fl	9.1-13
P-LCR	11.8	%	13-43
**Biochemistry**			
Fasting blood sugar	71	Mg/dl	70-106
Creatinine	0.9	Mg/dl	0.3-1.2
Sodium (Na)	141.8	mEq/l	135-148.5
Calcium (Ca)	9.1	Mg/dl	8.6-11.5

**Figure 4:**
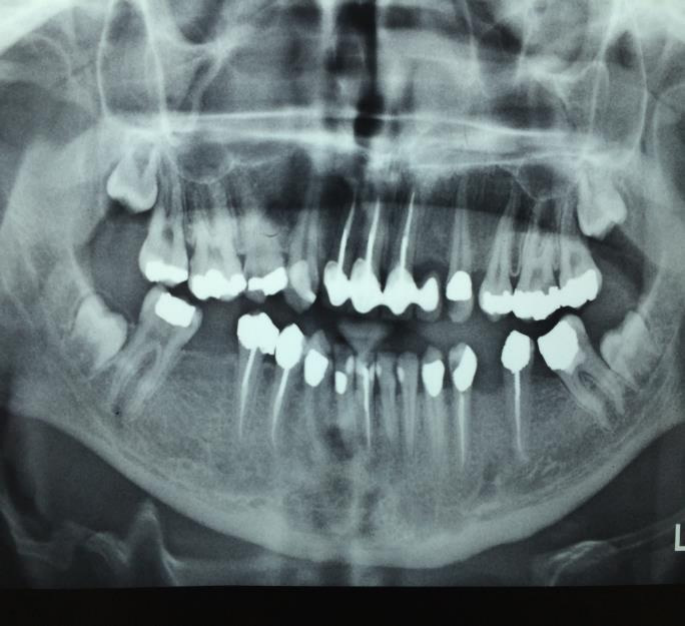
In panoramic radiography, because of hypoplastic enamel and poor oral hygine, all of teeth had feeling or need to endodontic treatments

The panoramic radiography showed that teeth had old filling or need endodontic treatment, because of hypoplastic enamel or intellectual disability and epilepsy with poor oral hygiene, of course for this patients, dentistry measures done under anesthesia in dentistry hospital (Figure 4).

**Figure 5:**
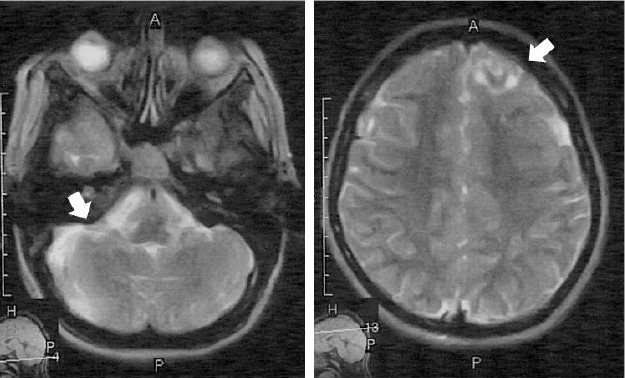
Cortical and subcortical tubers. In brain imaging, hypesignal foci are seen at subcortical white matter on long TR images. Fibers are involved. Considering the post history of tuberous sclerosis the most probable diagnosis is cortical and subcortical glioneuronal tubers

**Figure 6:**
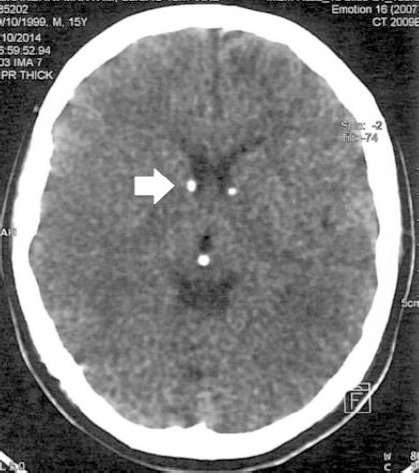
Some subepednymal glial nodules (white arrows) are also present in this case

Despite the physical examination showed absolutely intact neurologic examination, but brain MRI and CT scan (Figure 5 and 6) revealed several cortical and subcortical tubers, and subependymal glial nodules; no evidence of giant cell astrocytomas and aneurysm.

## 3. Discussion

The expression of the TSC varies substantially among individuals and within families (Crino et al., 2006; Schwartz et al., 2007; Curatolo et al., 2008). In this case, skin lesions were the most dominant presentation. The commonly reported lesions are hypopigmented macules, hypomelanotic lesions (ash leaf spot), wart-like lesions (facial angiofibroma) and fibromas; involve cells of neural crest (NC) origin. Previous studies speculated that these lesions may have a common root in NC (Sarnat & Flores-Sarnat, 2005; Weeks et al., 1994). As it is recalled from the embryology, the NC is a layer of multipotent cells sit between the neural or non-neural ectoderms and later immigrates and colonizes majority of embryo’s tissues. It derives to the verity of cells including central and peripheral nervous systems, glia, meninges, intracranial vessels’ smooth muscle cells, melanocyte, bone, cartilage and connective tissues of the skull (Dupin & Sommer, 2012). TSC cases should have a detailed skin examination at the time of diagnosis and annually thereafter. Disfiguring skin lesions may improve with laser therapy, dermabrasion, and possibly with topical mTOR inhibitors (Papadavid et al., 2002; Weinberger et al., 2009; Weiss & Geronemus, 2010).

International guidelines recommend performing a detailed dental and oral inspection or examination at the time of diagnosis to assess for dental enamel defects and intraoral fibromas (Krueger & Northrup, 2013). Thereafter, the guidelines recommend a dental and oral evaluation every three to six months, periodic preventive measures including oral hygiene, and panoramic radiographs by age seven years if not previously obtained. Dental pits are rarely symptomatic but can be treated with sealants if the patient is at increased risk of developing dental caries (Krueger & Northrup, 2013) oral fibromas that are symptomatic or interfering with oral hygiene can be surgically removed but may recur. Symptomatic or deforming jaw bone lesions should be treated with surgical excision or curettage. For other problems such as renal lesions, pulmonary diseases, to be refer to the physician (Crino et al., 2006; Schwartz et al., 2007; Curatolo et al., 2005).

In brain imaging, hypesignal foci are seen at subcortical white matter on long TR images. Fibers are involved. Considering the post history of tuberous sclerosis the most probable diagnosis is cortical and subcortical glioneuronal tubers. Some subependymal glial nodules are also present in this case. In our case, there is no evidence of giant cell astrocytomas and aneurysm.

Tubers rarely happen in the absence of subependymal nodules (SENs) (3%). Presence of SENs in absence of the tubers has been reported in only one case, suggesting that this phenotype occurs much rarely (Boronat et al., 2014). The various prevalence’s have been reported for SENs and tubers in different TSC studies, mostly related on which neuroimaging modality was used and the age of the studied population. Neuroimaging characteristics of SENs and tubers are different in infants younger than 3 months and patients aged >3 months (Datta et al., 2008; Baron & Barkovich, 1999). Because the intracranial future of infants younger than 3 months, cortical tubers are complicated and abnormalities of white matter are easier to be picked up in infants. SENs and white matter anomalies are shown hyperintense on T1-weighted MRI images and become hypointense on T2-weighted MRI images. However the opposite pattern is seen in older patients. As always the wide verity of the populations including infants and adults have been studied by CT or MRI, this could be the probable explanation that why some studies reported a higher rate of tubers (Webb et al., 1991; Michelozzi et al., 2013), while other reports showed higher rates of SENs (Dabora et al., 2001; Sancak et al., 2005), or same frequencies of both lesions (Braffman et al., 1992; Au et al., 2007).

Retinal hamartoma was not shown in this case and although the retinal epithelium originates from the neural ectoderm (Schoenwolf et al., 2008). Cardiac rhabdomyomas, lymphangioleiomyomatosis and angiomyolipoma were also absence in our case, despite the fact that the NC plays a role in development of the heart in forming the septum, bifurcating the truncus into the aorta and pulmonary arteries root and also developing to the connective tissue which turn into the His-Purkinje conduction system (Huston & Kirby, 2007). Lung, kidney, sclerotic bone lesions and uterine leiomyomas were not found in this TSC case.

The outstanding characteristic of this case was her normal kidney, heart, and lung (which are usually abnormality of these organs in TSC cases leads to death) except seizure, intellectual disability, skin lesions and dental enamel involvement. However, she has not oral mucosa involvement. This case referred to the hospital due to the seizures and intellectual disability although she does not have any signs and symptoms in organs of body. 7 years after that, skin lesions appeared. Thus, it seems that TSC could be the prevalent disorder and referring intellectual disability patients in birth with normal organs could be diagnosed as TSC. Therefore, there is necessary need to design genetic natal and post natal tests for the diagnosis of TSC cases (like Down syndrome). Also, there is pivotal that similar cases must be reported; perhaps TSC is more prevalent than to be considered.
